# Do family and neighbourhood matter in secondary school completion? A multilevel study of determinants and their interactions in a life-course perspective

**DOI:** 10.1371/journal.pone.0172281

**Published:** 2017-02-21

**Authors:** Arnhild Myhr, Monica Lillefjell, Geir Arild Espnes, Thomas Halvorsen

**Affiliations:** 1 NTNU Center for Health Promotion Research, Department of Neuromedicine and Movement Science, Faculty of Medicine and Health Sciences, Norwegian University of Science and Technology, Trondheim, Norway; 2 NTNU Center for Health Promotion Research, Department of Public Health and Nursing, Faculty of Medicine and Health Sciences, Norwegian University of Science and Technology, Trondheim, Norway; 3 SINTEF Technology and Society, Department of Health Research, Trondheim, Norway; TNO, NETHERLANDS

## Abstract

**Background:**

Completion of secondary education is important for individuals’ future health and health behaviour. The fundamental purpose of this study is to investigate the variation and clustering of school completion in families and neighbourhoods. Secondly, we aim to examine the impact of individuals’ family structure and neighbourhood of residence and examine to what extent parental education level moderates these associations.

**Methods:**

Longitudinal register data for 30% of the entire Norwegian population aged 21–27 years in 2010 (N = 107,003) was extracted from Statistic Norway´s event database. Three-level logistic regression models, which incorporated individual, family, and neighbourhood contextual factors, were applied to estimate the family and neighbourhood general contextual effects and detect possible educational differences in the impact of family structure and urban place of residence in school completion.

**Results:**

Completion rates were significantly higher within families with higher education level (79% in tertiary educated families vs. 61% and 48% in secondary and primary educated families respectively) and were strongly correlated within families (ICC = 39.6) and neighbourhoods (ICC = 5.7). Several structural factors at the family level negatively associated with school completion (e.g., family disruption, large family size, and young maternal age) were more prevalent and displayed more negative impact among primary educated individuals. Urban residence was associated with school completion, but only among the tertiary educated.

**Conclusions:**

Investment in the resources in the individuals’ immediate surroundings, including family and neighbourhood, may address a substantial portion of the social inequalities in the completion of upper secondary education. The high intra-familial correlation in school completion suggests that public health policies and future research should acknowledge family environments in order to improve secondary education completion rates among young people within lower educated families.

## Introduction

Completion of upper secondary education strongly influences future health and health behaviours [1, 2]. Thus, policies that promote school completion have the potential to improve health disparities. In Norway, 30% of students do not complete upper secondary education, and, as in many other western societies, non-completion is a major concern. The level of education affects health and happiness, standard of living, and socioeconomic status (SES) [3–6]. Educated people are more likely to succeed in the labour market [7], are more actively engaged in society, and tend to make better choices about factors that affect their health and quality of life [1, 2, 4, 8].

A large body of research shows that early contextual exposures such as family origins and neighbourhood have long-term implications for individuals’ life courses [9–12]. The life course perspective emphasizes the understanding of how early life experiences can impact health in the course of life and potentially across generations. Attention is systematically given the role of context, including social and physical context, along with biological factors over time [13]. Material and psychosocial factors experienced in childhood encompass many important aspects of individual health later in life, including the potential for later educational attainment, and for the socio-economic circumstances of adult life [14]. According to the life-course perspective, the accumulated effects of advantages and disadvantages across the lifespan determines the social distribution of health and illness [9]. However, children have very different departure points depending on their parents’ characteristics [8]. Elder et al. [15] propose that young individuals construct their own life paths through their choices and actions, but within the context of historical and social conditions [15–18].

Life course research suggests that family and social environment during the early years of life strongly predict a child’s health and academic success [19–22]. To a large extent families share SES and other social determinants of health and transfer them between generations [10, 11, 23]. Thus, children born into high-SES families tend to achieve high SES in adulthood. The family and its resources and strategies affect the offsprings' exposure to a wide range of physical and psychosocial conditions and for this reason is considered among the most powerful social contexts fostering successful outcomes such as educational achievement [19–22]. A large Norwegian population study found a strong association between school non-completion and parental SES, with strong clustering at the family level—that is, family level determinants account for a large proportion of the variation (42%) in school dropout rates [24]. Moreover, educational achievement correlates strongly with parental SES [25, 26] and family adversity such as parental divorce [27, 28], unemployment [25], and poverty [26]. Sibling compositions, especially in terms of family size and birth order, also influence educational outcomes [29–31].

Complicating the findings of the large populations study, environmental factors are not randomly distributed among families [32]. Adverse circumstances in one context often causes less favourable conditions in other contexts as well [9]. Poorly educated individuals, for example, suffer more often from lower average income, higher unemployment, and higher reliance on public benefits than their peers [33–35]. They also tend to have more children and are more likely to be single parents. However, family SES is not only important in influencing individuals' own adulthood attainment through the transmission of values and life expectations. Resources also affect individuals' exposure to a wide range of physical and psychosocial conditions during childhood, as the socioeconomic position of the household is also correlated with neighbourhood of residence and the school environment [36]. Numerous different neighbourhood factors have been put forward as determinants of educational achievement [12, 37]. Deprived neighbourhoods suffer from the clustering of social problems including low education level of adults, unemployment, high rates of receipt of public benefits, poor health, and low educational achievement of children [12]. Similarly, people living in rural areas are underrepresented in the tertiary-educated population and the educational performance of rural children is generally lower than that of their urban counterparts [38]. In the past twenty years Norway has undergone a large geographic centralization, which has led to a dramatic rise in house prices in major cities [39]. Because of this, less expensive house prices in rural areas attract population groups with lower socioeconomic profiles.

Hopefully it is apparent, given the preceding discussion, that human beings are part of a larger entity with powerful influences at multiple levels of society that change over time. A number of mechanisms linking childhood family SES to school performance have been proposed, most of which involve differences in access to material and psychosocial resources or responses to stress-inducing conditions for both the parents and their children [26]. However, whether the stark inequalities in health and its social determinants are rooted in absolute or relative material standards is under great debate. In general, wealthy people enjoy better material living standards, which in turn are found to positively influence their health and educational achievement [40, 41]. However, in rich countries with welfare states, such as Norway, there is less relation between average income and life expectancy, indicating that health is also related to relative inequality [42]. According to the relative SES hypothesis [43, 44] the disparity between individuals' own socioeconomic position and the socioeconomic position of those living nearby affect individual health. The Whitehall studies of British civil servants by Marmot and his colleagues [45–48] demonstrated that social inequalities in health also exist among those who are not poor. In other words, poor individuals living in wealthy areas may experience more negative health effects than people of similar SES status whose neighbours share their status [49–51]. Social distance, distrust, and lack of cohesion between population groups typically characterise societies with high material and social inequalities [52]. This may lead to higher stress levels, especially for those at the bottom of the social ladder [53, 54].

It is evident that all people, some more and others less, are influenced by our surroundings. However, how and how much these contexts influence us is dependent on several factors including personality, individual psychosocial risk factors, and our sensitivity both to the immediate social environment and to the broader social structures in the modern society [26, 55]. As previously mentioned, negative development outcomes such as non-completion of secondary education is conditioned by family origin and parenteral socioeconomic resources, which in turn influence a range of other physical and psychosocial conditions known to be related to educational achievement. In other words, an individual’s propensity to complete secondary education may be connected to family SES, particular SES cofactors (such as single parenthood, neighbourhood deprivation, or school environment), some combination of these factors, or even a third factor connected to both family SES and SES cofactors (e.g., family conflict, residential mobility) [26]. Consequently, a single socioeconomic context may have very different impacts at both the family and individual levels. Thus, adjusting for potential important contextual levels is both necessary and of substantial interest.

A large body of literature has recognized the importance of family origins and neighbourhood in educational achievement [56], but so far there has been little research on how parental education levels moderate these associations. By exploring educational differences in the impact of family structure and neighbourhood of residence, the current study seeks to identify critical factors that might prevent school non-completion among particular subgroups of the population. As such it begins to illuminate how the various components of SES interact synergistically to affect the course of development, a significant benefit to creating targeted measures and policies to improve school completion rates.

### Aims and hypotheses

The fundamental purpose of the present study is to investigate the variation and clustering of completion of upper secondary education in families and neighbourhoods (i.e., the degree to which family origin and neighbourhood of residence affect individual variance in school completion). Secondly, we aim to analyse the impact of family structure and neighbourhood on school completion and examine to what extent parental education level moderates any of the hypothetical associations. Based on the existing literature, which shows that family structure and neighbourhood contexts influence the probability of completing secondary education, and that these associations may vary across different socioeconomic groups [9, 57, 58], we hypothesize:

1. Family disruption, having many siblings, and having a young mother (<20 years of age at birth) are disadvantageous family structures that negatively affect the probability of school completion (H1).
2. Parental education level moderates the relationships with family structure and completion of upper secondary education (H2).
3. An urban place of residence increases the probability of completing secondary education (H3).
4. Parental education level moderates the relationship between urbanity and completion of secondary education (H4).

## Methods

### Data sources

**Fig 1:**
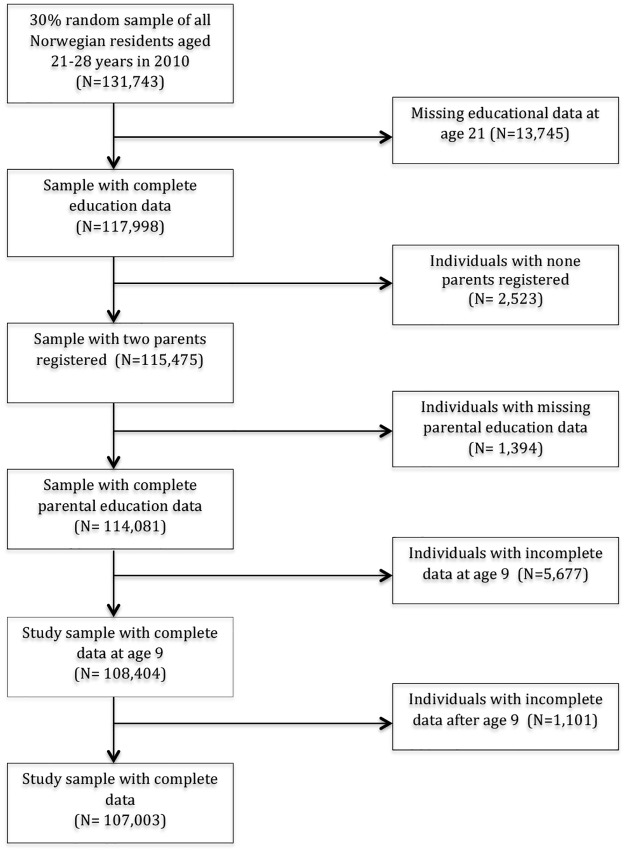
Flow chart of the participants in the present study who were included in the analyses.

Statistics Norway’s event database (FD-Trygd) [59] and the Norwegian National Education Database (NUDB) [60] supplied us with national administrative data for the period 1992–2010. The FD-Trygd database assembles event registration data from several official administrative and statistical registers and includes life cycle events in demography, work status, income, and different types of national insurance statuses. FD-Trygd covers all Norwegian citizens and contains information from 1992 and onwards. We extracted a random sample, stratified by age, gender, and municipality of residence, for 30% of the entire Norwegian population aged 21–27 years in 2010 (i.e., born in the period 1983–1989, N = 107,003). This cohort gave us long enough follow-up periods to determine the effect of measures of childhood family and neighbourhood context on completion of upper secondary education. We merged this dataset with the NUDB database by using the unique 11-digit personal identification numbers assigned to all Norwegian citizens. Through a unique family identification code attached to each personal identification number, we were able to allocate information on the parents and the household to each individual. This enabled us to map the background of the parents and to determine whether the individual lived with his or her parents. Hence, we ended up with linked longitudinal data for both subjects and their parents, including annual updates on demographic, socio-economic information, and factors related to family structure. We excluded 13,745 individuals (7.5%) from the sample (of which 97% had immigrant backgrounds) due to missing their educational data at age 21. (See Fig 1 for inclusion and exclusion criteria for the study sample.) The resulting dataset contained 107,103 individuals.

### The outcome variable

Our dependent variable is whether or not the individual completed upper secondary education by age 21. NUDB provided this information. In Norway young people generally begin upper secondary education at 16, and it consists primarily of a high school academic track of three years and/or vocational education, which lasts between 2 and 4 years [61]. We examined completion rates 5 years later, i.e., at age 21. Official statistics show that 70% of all Norwegian citizens complete upper secondary education within a 5-year period [62].

### Explanatory variables

#### Individual level

At the level of individuals, the data included information on gender and teenage parenthood. Several studies have documented that teenage parenthood reduces the probability of completing secondary education [63]. Additionally, completion of secondary education displays gender differences, with higher completion rates among females [62].

#### Family level

The unit of analysis at the second level is the families (N = 99,945) identified in the study sample. In our dataset, we directly observe family members in the time period 2005 to 2010 through a unique family identifier obtained from SSB’s event database. The family identifier connects all members of a family, which consists of persons who are registered as living in the same household and are related to each other as spouses, registered partners, cohabitants, and/or parent and child (regardless of the child’s age). In the time period 1992–2004, no family identifier was available. However, we were able to identify siblings who shared the same mother and father during this time period by matching the parents’ unique personal identifiers, which identifies parental relationships by linking parents to children.

Parental education level, obtained from the educational registers of SSB, was based on the Norwegian standard classification of education [60], providing nine levels which were collapsed into three education level groups: primary, secondary, and tertiary education. The indicated parental education refers to the parents with the highest educational level. Previous studies have found that education level reliably predicts SES in mid-life [64]. Mercy and Steelman [65] found that family income and maternal and paternal education predicted academic attainment, with parents’ education being the best predictor.

We used several variables to control for the impact of childhood “family structure,” including family living situation, number of siblings, and mother’s age at birth. Family living situation was categorized into five categories, defined as living with (1) two registered parents both at age 9 and 16, (2) two parents at age 9 and one parent at age 16, (3) one parent at age 9 and 16, and (4) not living with parents at age 16. The number of siblings was categorized as only child, 2–3 siblings, or more than 3 siblings. Maternal age at birth was categorized as 1) <20 years, 2) 20–30 years, and 3) >30 years. Additionally, the analysis was adjusted for the dichotomous variables of parental employment and poverty. “Employed” was defined as being registered as employed in SSB’s event database at the index person’s age 9, and the parent had to be registered as employed for more than half of a calendar year to be categorised as employed. Poverty was defined as having parents receiving social security benefits in the period 9 to 16 years of age (according to the index person’s age).

Household income, derived from the tax registries, was available only for the period 2003–2010. Thus, this variable was a valid measure of family income only for the 1987–1989 cohorts. We aggregated the last non-missing household income to the individual level using the personal identification number. In the original annual data the top 3% of the highest incomes was collapsed to give them the same value. The household income was kept continuous and used only in a supplementary analysis containing the 1987–1989 cohorts (results not shown).

#### Neighbourhood level

The unit of analysis at the third level is neighbourhood. We use the individuals' recorded census enumeration district, which is the lowest geographical level for population statistics, to identify their neighbourhoods [59]. Neighbourhood of residence was measured at age 9. The dichotomous variable “urban” identifies the neighbourhood of residence as urban or rural according to the FD-Trygd database. Urban settlements have clusters of homes where at least 200 people live within a distance of 50 meters or less; rural areas are defined as having a lower population density than this threshold [66].

### Statistical methods

We investigate the relationship between completion of secondary education, parental education, family structure, and neighbourhood of residence, testing the hypothetical interaction and its possible mediators by using three-level logistic regression analysis. Individuals (level 1, n = 107,003) are nested within families (level 2, n = 99,945), which is nested within neighbourhoods (level 3, n = 11,179). Each of these contexts may condition individual level variation due to unmeasured factors. Hence, we fitted a three-level random intercept model [67–69] to distinguish the individual, family, and neighbourhood sources of variation in the completion of upper secondary education. The model can be expressed as

$$\text{log}it(p_{ijk})=log\left[p_{ijk}/\left(l-p_{ijk}\right)\right]\;=\beta_{a}+\beta_{b}x_{bk}+\beta_{c}x_{cjk}+\beta_{d}x_{dijk}+\beta_{e}x_{bk}x_{cjk}x_{dijk}+u_{k}+u_{jk}+e_{ijk}$$

where p_ijk_ is the probability of completing upper secondary education for individual i in family j within the neighbourhood k;. u_jk_ and u_k_ denote the second level (family) and the third level (neighbourhood) random effect factors which are the log odds differences that follows a normal distribution; *β* is a model coefficient to be estimated; and x_ijk_, x_jk_, and x_k_ represent a set of explanatory variables at the individual, family, and neighbourhood levels, respectively. u_jk_ remains constant for individuals within a family but varies across families and neighbourhoods. Similarly, u_k_ is constant for families in a neighbourhood but varies across neighbourhoods.

The multilevel statistical modelling framework allows the simultaneous examination of the effects of group-level and individual-level predictors while also accounting for non-independence of observations (clustering) within higher level units. For the present study this framework allows the estimation of both specific contextual effects (i.e., the association between a particular family or neighbourhood characteristic and individual propensity for school completion) and general contextual effects (i.e., the degree to which the family and neighbourhood context, as a whole, conditions individual variance in school completion).

**Fig 2:**
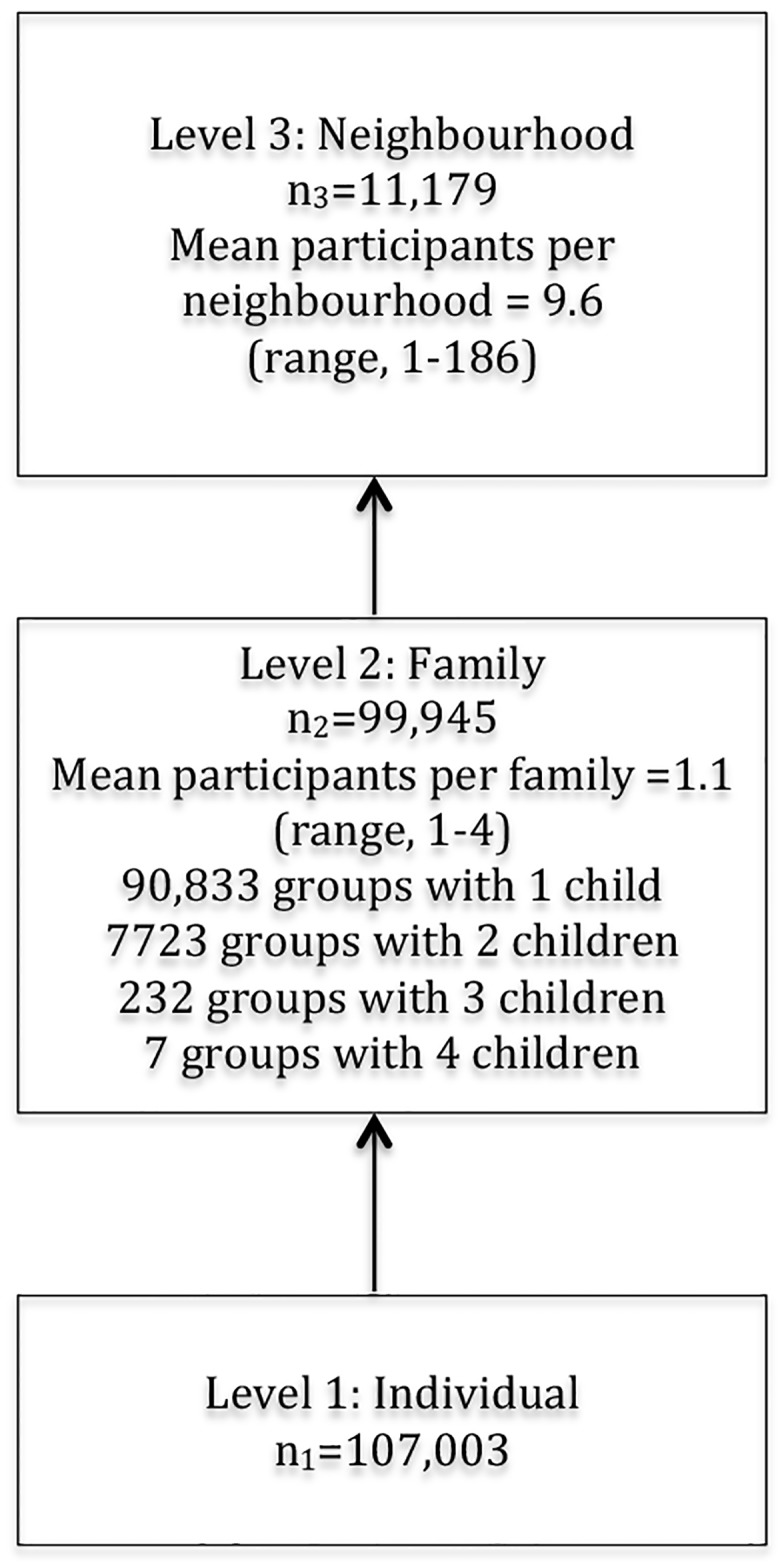
Diagram for the 3-level classification model of individuals nested within family groups and family groups nested within neighbourhoods.

We modelled the prediction of school completion in seven steps. First, we estimated an “empty” model, which includes only a random intercept, representing the variation in outcome between the three initial levels. This allowed us to determine the impact of the family and neighbourhood context on the outcome [70]. Model 1 included all the variables (with no interaction effects) including parental education level, family structural variables (i.e., family living situation, siblings, and maternal age) and urban residence, adjusted for the individual variables. In Model 2 we adjusted for family socioeconomic resources (i.e. parental employment and poverty). Models 3–5 added the interaction terms parental education to family living situation (Model 3), maternal age (Model 4), and urbanity (Model 5). In the final model (Model 6), all variables including the interactions terms with parental education level were included. The interaction of parental education and sibling size was not significant when adjusted for the family socioeconomic resources and was thus not included in the final model. In the 1987–1989 cohort we also adjusted for household income (results not shown). To investigate the combined effect of two well-known risk factors for non-completion, low parental education level and parental unemployment [25], we used interaction analyses with a synergy index (S) as the measure [71]. A synergy index above one (S>1) indicates the two exposures (e.g., low education level and unemployment) act jointly, implying a stronger effect of one exposure in the presence of the other. The S4 and S5 Tables show the results from the calculations. To estimate the family level variance we need to have multiple children per family [72]. Since most individuals in the present study were in family groups of only one child (see Fig 2), the variance at the family level for these individuals included the individual variance. To account for this in the analysis, we estimated the family variance only for those families in the study sample with more than one child [72, 73]. Estimates for fixed effects are reported as odds ratios (OR) with 95% confidence intervals (95% CI). The main focus in the analysis was on variance and consequently we assessed the relative importance of the general contextual effects of neighbourhoods and family groups by the following measurements: (a) variance (on the log odds scale) with 95% CI, (b) the median odds ratios (MOR) [70] and (c) the intra class correlation coefficients (ICCs) [69]. The MOR translates the variance estimated on each level on the log-odds scale and may in our case, in a simplified way, be interpreted as the increased median odds of completing secondary education if an individual were living in another family (or neighbourhood) with higher risk [74]. Thus, the higher the MOR, the greater the general contextual effect. ICC may be interpreted as the correlation in the outcome (school completion) between two individuals taken randomly from the same family (or neighbourhood). By using the latent variable approach [69, 75] that considers the variance from a standard logistic distribution (π^2^/3 = 3.29), we calculated the ICC as the proportion of variance on a given contextual level divided by the total variance. Consequently ICC was calculated as:

For level 3 (neighbourhood): V_level-3_/ (V_level-3_+ V_level-2_+3.29)
For level 2 (family): (V_level2_+V_level3_)/ (V_level2_+V_level3_+3.29)

We also calculated the percentage of proportional change in variance (PCV) in order to quantify the proportion of family and neighbourhood level variance of the empty model explainable by predictor variables input into the more complex models. The PCV is calculated as

$$PCV=\frac{\left(V_{A}-V_{B}\right)}{V_{A}}$$

where V_A_ is the variance at the particular level of the initial model, and V_B_ is the model with more terms.

The multilevel regression model parameters were estimated by using mixed effects logistic regression as implemented in STATA/MP software (version 13). We performed secondary analyses, only containing families in the study sample with more than one child, in order to estimate the family level variance. These analyses was performed by using both mixed models in Stata and Markov chain Monte Carlo (MCMC) methods as implemented in the MLwiN multilevel modelling software, version 2.32 [76] in order to check robustness of the family level variances. For the latter, after obtaining maximum likelihood estimates as starting values, we specified a burn-in length of 10,000 iterations and a monitoring chain length of 50,000 iterations for each model. For the final model we specified a monitoring chain length of 500,000 iterations. Visual assessments of the parameter chains and standard MCMC convergence diagnostics suggest that the lengths of these periods were sufficient. For the fixed effects, non-informative Gaussian priors were used, and non-informative uniform priors for the between-family variances. The Bayesian deviance information criterion (DIC) was used as a measure of goodness of fit of our models [77]. The DIC considers both the model deviance and its complexity. Models with smaller DIC are preferred to models with larger DIC [78].

### Ethics statement

Statistic Norway constructed the study sample with linked longitudinal data for both the subjects and their parents, by means of record linkage of different registries integrated into the SSB database by using the unique Norwegian personal identification number. Finally, Statistic Norway delivered the data to us without personal identification numbers to ensure the anonymity of the study subjects. The Regional Committee for Medical and Health Research Ethics (REK) of Mid-Norway (permission 2011/783) approved the study and the data linkage procedures.

## Results

### Descriptive statistics

Table 1 presents descriptive information for the individual childhood variables as well as recorded completion of secondary education within the follow-up period in the three family education groups. Completion rates were significant higher within families with higher education level: 82% in tertiary educated families vs. 67% and 56% in secondary and primary educated families, respectively. Moreover, individuals whose parents held a tertiary education more often lived with both parents during childhood and had older mothers than individuals whose parents had lower levels of education. Additionally, tertiary educated parents had higher employment rates and were less likely to live in poverty compared with primary and secondary educated parents.

**Table 1:** Individual, family, and neighbourhood characteristics according to parental educational level among individuals born in the period 1980–1989.

Predictors	Primary	Secondary	Tertiary	P-value^c^	Start^e^	Stop^f^
	(N = 24,228)	(N = 37,424)	(N = 45,351)			
	%	%	%			
**Individual level**^a^						
Completed secondary school	55.9	67.2	81.7	0.000	1992	2010
Female	48.9	48.6	48.9	0.709	1992	2010
Teenage parent	2.9	2.1	0.9	0.000	1992	2010
**Family level**^b^						
Number of siblings				0.000	1992	2010
Only child	18.6	13.7	11.9			
2–3 siblings	71.6	79.4	79.9			
More than 3 siblings	9.8	6.9	8.2			
Family living situation				0.000	1992	2010
Two parents at age 9 and age 16	62.1	67.5	73.7			
One parent at age 9 and age 16	10.3	10.9	9.9			
Both parents at age 9, one at age 16	25.1	20.7	16.0			
Not living with parents at age 16	2.4	0.9	0.4			
Maternal age				0.000	1992	2010
<20	6.1	5.6	2.1			
20–30	66.6	76.9	64.6			
30+	27.2	17.5	33.4			
Parental employment				0.000	1992	2010
Both parents in work	50.5	62.6	71.9	0.000	1992	2010
One parent in work	36.9	32.5	25.0			
None parents in work	12.6	4.9	3.2			
Poverty	29.2	17.6	8.7	0.000	1992	2010
Household income (mean, SE)^d^	619.3(8.25)	700.6(7.26)	940.9(18.07)	0.000	2003	2010
Only one parent registered	2.5	0.8	0.7	0.000		
**Neighbourhood level**^a^						
Urban	69.4	70.6	80.7	0.000	1992	2010

^a^ Statistics based on the total study sample.

^b^ Statistics with only one random respondent from each family group.

^c^ P-value for the comparisons between the education groups (Pearson chi square test and ANOVA).

^d^ Measured in thousands, only for the 1987–1989 cohort.

^e^ Data available from this year.

^f^ Data available until this year.

### Do family and neighbourhood of residence matter?

The ICC and MOR calculated from the variances are fairly high for the empty model at the family level (ICC = 39.6, (MOR = 3.90) and the neighbourhood level (ICC = 5.7, MOR = 1.48), although the neighbourhood variance is much smaller than the individual and family level variances. This indicates that the underlying causes of the completion of secondary education have a systematic family and neighbourhood variation. It also suggests that further exploration of the causes of the between-family and -neighbourhood variation in multi-level models is both necessary and of substantial interest. The family- and neighbourhood-level variance decreased substantially when the model included parental education level, family structural variables, urban vs. rural residence, and the interactions terms (Models 1–6).

#### The impact of parental education, family structure and their interaction on the completion of secondary education

Models 1–2 in Table 2 display the observational associations with school completion and individual characteristics, parental education level, family structural variables, and urban vs. rural residence (Model 1), adjusted for family socioeconomic resources (parental employment and poverty, Model 2). As a group, these variables explain a major part of the individual variation in the completion of upper secondary education. The association with parental education level is strong: high education background more than quadruples the individual’s chances for completing upper secondary education. “Family structure” affects the probability of completing secondary education. Children living in two-parent families at both age 9 and age 16 have a significantly higher probability of completing secondary education compared to those who experience family disruption during childhood (OR = 0.42) and those who live with only one parent most of their childhood (OR = 0.32). Moving away from parents before turning 16 was associated with the highest risk for not completing secondary education (OR = 0.13). Having more than three siblings was negatively associated with completion (OR = 0.83), although this association disappears when adjusting for parental employment and poverty. The results from the analysis strongly support our first hypothesis, namely that low parental education level, family disruption, large family size, and low maternal age reduce the probability of completing secondary education.

**Table 2:** The effects of parental education level, family structure and neighbourhood of residence, and its interactions on the probability of completing secondary education at age 21 among individuals born in the period 1983–1989.

	Model 1		Model 2		Model 3		Model 4		Model 5		Model 6	
	OR	95% CI	OR	95% CI	OR	95% CI	OR	95% CI	OR	95%CI	OR	95%CI
**Fixed effects**												
**Individual level**												
Female	1.99	1.91–2.07	1.98	1.90–2.06	1.97	1.89–2.06	1.98	1.90–2.06	1.98	1.90–2.06	1.97	1.89–2.06
Teenage parent	0.08	0.07–0.10	0.09	0.08–0.10	0.09	0.08–0.10	0.09	0.08–0.10	0.09	0.08–0.10	0.09	0.08–0.10
**Family level**												
Family education level												
Primary			Ref		Ref		Ref		Ref			
Secondary	1.77	1.68–1.86	1.58	1.50–1.66	1.60	1.51–1.70	1.51	1.37–1.66	1.48	1.36–1.61	1.44	1.27–1.62
Tertiary	4.22	3.95–4.51	3.44	3.23–3.66	3.69	3.44–3.97	3.23	2.94–3.55	3.03	2.27–3.35	2.97	2.63–3.37
Siblings												
Only child	Ref		Ref		Ref		Ref		Ref		Ref	
2–3	1.08	1.02–1.13	1.09	1.03–1.15	1.08	1.03–1.14	1.09	1.03–1.15	1.09	1.03–1.15	1.08	1.03–1.14
4+	0.83	0.77–0.90	1.00	0.92–1.08	0.99	0.92–1.07	1.00	0.93–1.08	1.00	0.93–1.08	1.00	0.92–1.08
Family living situation												
Two parents at age 9 and 16	Ref		Ref		Ref		Ref		Ref			
Both parent at age 9, one at age 16	0.42	0.40–0.45	0.52	0.49–0.55	0.59	0.53–0.66	0.52	0.49–0.55	0.52	0.49–0.55	0.59	0.53–0.67
One parent at age 9 and at age 16	0.32	0.31–0.34	0.50	0.47–0.53	0.53	0.49–0.58	0.50	0.47–0.53	0.50	0.47–0.53	0.53	0.49–0.58
Not living with parents at age 16	0.13	0.11–0.16	0.27	0.23–0.33	0.32	0.25–0.41	0.27	0.23–0.33	0.27	0.23–0.33	0.32	0.25–0.41
Maternal age at birth												
<20	0.42	0.39–0.47	0.53	0.48–0.58	0.53	0.48–0.58	0.61	0.52–0.72	0.53	0.48–0.58	0.60	0.51–0.71
20–30	0.83	0.79–0.87	0.86	0.82–0.90	0.86	0.83–0.90	0.80	0.74–0.86	0.86	0.82–0.90	0.80	0.73–0.86
30+	Ref		Ref		Ref		Ref		Ref		Ref	
Only one parent registered	0.93	0.79–1.08	0.85	0.72–0.99	0.86	0.73–1.01	0.85	0.73–1.00	0.85	0.72–0.99		
**Neighbourhood level**												
Urban settlement	0.94	0.90–0.99	0.97	0.93–1.02	0.97	0.93–1.01	0.97	0.93–1.02	0.89	0.83–0.96	0.89	0.82–0.96
**Socioeconomic controls**												
Parental employment												
Both parents in work			Ref		Ref		Ref		Ref		Ref	
One parent in work			0.77	0.74–0.80	0.77	0.74–0.81	0.77	0.74–0.80	0.77	0.74–0.80	0.77	0.74–0.81
None parents in work			0.65	0.60–0.70	0.65	0.60–0.70	0.65	0.60–0.70	0.65	0.60–0.70	0.65	0.60–0.70
Poverty			0.42	0.39–0.44	0.42	0.39–0.44	0.42	0.39–0.44	0.42	0.39–0.44	0.42	0.39–0.44
**Interactions with parental education level**												
Family education level*living situation												
2*Two parents at age 9, one at age16					0.88	0.76–1.01					0.87	0.76–1.01
2*One parent at age 9 and at age 16					1.02	0.92–1.14					1.02	0.91–1.14
2*Not living with parents at age 16					0.78	0.53–1.16					0.79	0.53–1.16
3*Both parent at age 9, one at age 16					0.81	0.70–0.94					0.80	0.69–0.93
3*One parent at age 9 and 16					0.82	0.73–0.91					0.81	0.72–0.91
3*Not living with parents at age 16					0.66	0.42–1.06					0.67	0.42–1.07
Family education level*maternal age												
2 *<20 years							1.09	0.98–1.21			1.09	0.97–1.21
2 *20–30							0.83	0.68–1.03			0.83	0.67–1.03
3 *<20 years							1.12	1.02–1.24			1.15	1.04–1.27
3 *20–30							0.71	0.56–0.91			0.78	0.61–1.00
Family education level*Urban												
2 *Urban									1.10	0.99–1.21	1.09	0.99–1.21
3 *Urban									1.19	1.06–1.32	1.21	1.08–1.34
Random effects												
**Neighbourhood variance (95% CI)**	0.13	0.11–0.16	0.11	0.09–0.13	0.11	0.08–0.13	0.11	0.08–0.13	0.11	0.08–0.13	0.10	0.08–0.13
PCV	-60.6%		-66.7%		-66.7%		-66.7%		-66.7%			
ICC(%)	2.7		2.23		2.23		2.23		2.21		2.21	
MOR	0.94		0.86		0.86		0.86		0.86			
**Family variance (95% CI)**	1.43	1.18–1.74	1.34	1.10–1.63	1.31	1.07–1.61	1.34	1.10–1.63	1.34	1.10–1.63	1.31	1.07–1.61
PCV^a^	- 35.9%		- 39.9%		- 41.3%		-39.9%		- 39.9%			
ICC (%)	29.5		28.3		27.9		28.3		28.3		27.8	
MOR	3.09		2.99		2.96		2.99		2.99			
Family variance >1 child*												
**Family variance (95% CI)**	1.49	1.21–1.84	1.37	1.10–1.70	1.36	1.09–1.69	1.37	1.10–1.70	1.37	1.10–1.70	1.36	1.09–1.69
** PCV**	-34.7%		- 39.9%		- 40.4%		- 39.9%		- 39.9%		- 40.4%	
** ICC (%)**	30.6		29.0		28.8		29.0		29.0		28.8	
** MOR**	3.15		3.02		3.01		3.02		3.02		3.01	

* Family level variance from the secondary analysis containing only families with more than one child (S1 Table)

^a^ The proportional change in variance expresses the change in variance at the particular level from the empty model

Models 3–6 examine whether parental education level moderates the associations with family structural variables. The interactions between parental education and family structure had the predicted effects and significance levels. In other words, the results support our second hypothesis. Among individuals with tertiary educated parents the negative impact of family disruption and leaving home at an early age becomes weaker (Table 2, Model 3). The negative impact of having more than two siblings becomes positive among the tertiary educated families, although this disappears when the models are adjusted for family socioeconomic factors (results not shown). The negative association with young maternal age at birth was present in all three education groups, but was, however, less significant among individuals with tertiary educated parents. Notably, a maternal age of 20–30 was associated with the highest propensity to complete school among the tertiary educated group, whereas among primary and secondary educated a maternal age of 30+ was associated with the highest risk. Increasing levels of parental education seems to dampen the impact of adverse family structural variables. Significantly, even individuals with tertiary educated parents still showed such an impact.

#### The impact of urban settlement and its interaction with parental education on the completion of upper secondary education

Models 1, 2, and 5 further test the rural-urban dimension of the outcome. Contrary to our third hypothesis, we found that urban settlement is associated with lower odds for completion of secondary education (OR = 0.94), although this association disappears when adjusting for parental employment and poverty (Model 2). However, Model 5 shows that the interaction term with parental education level is significant, indicating that the impact of urban residence differs among the education groups. Among individuals of tertiary educated parents urban settlement is actually associated with completion (OR = 1.19). These associations were also stable when adjusting for all the other variables in the model (Model 6). Parental employment and household income (available only for the 1987–1989 cohorts, results not shown) were positively associated with completion, whereas family poverty showed the opposite relationship.

## Discussion

The key contributions of this study are related to the exploration of how family structure and neighbourhood of residence influence the probability of completing upper secondary education among young Norwegians, and to the clarification of to what extent parental education level moderates these associations. This study deepens our understanding of how different dimensions of SES interact with each other, and with childhood family and neighbourhood contexts, to facilitate or impede educational achievement in young adulthood.

Our results show that the probability of completing upper secondary education is strongly clustered within families and moderately clustered within neighbourhoods. As prior research predicts [26], we found a strong relationship between secondary school completion and parental education level. Furthermore, disadvantageous family structural conditions such as family disruption, large family size, and young maternal age reduce the propensity to complete secondary education. However, our analysis shows that the parental education level moderates the impact of these associations. Moreover, urban settlement was associated with school completion, but only among individuals originating from higher education levels. These findings, in combination with the fact that the intra-familial correlation in individual school completion risk was much higher (ICC = 39.6%) than the intra-neighbourhood correlation (ICC = 5.7%), suggest that there is a need for preventive family level interventions particularly aimed at families of lower education level. It is, in other words, important to consider whether measures to improve completion rates should be specified according to family education background. Health behaviour or living habits affecting health differs with education level [1, 4, 8, 9]. Hence, preventive measures to improve completion rates may have a positive impact among individuals from families with higher education levels, but a negative impact among their peers from lower educated families. Educational differences in health behaviour may reflect greater resources, better opportunities, greater ability to absorb relevant health information, and more efficient use of health services among those with higher education. In practice, this may have the paradoxical outcome that preventive measures reinforce social inequalities in health because the highly educated tend to make better choices about factors that affect their health and quality of life.

Elder’s life course theory [15, 16] proposes that individuals construct their own life course through their choices and actions, but within the constraints of historical and social circumstances. Hence, secondary education completion cannot be understood without taking into account the social structure and culture of the childhood living environment. The present study found that family origin and neighbourhood of residence are important factors in upper secondary education completion. In agreement with previous literature [2, 9, 25], we found that the probability of completing upper secondary education systematically increased with parents’ education level. According to Helland and Støren [79], parental education level affects social differences in education by facilitating educational aspirations, values, preferences, and achievements. Furthermore, disadvantageous circumstances in one context often amplify adverse conditions in other contexts [9]. Our findings reflect this amplification as well. Several of the adverse family structural factors, including family disruption, large family size, and young maternal age were more prevalent and had stronger negative impact among adolescents from families with low education. Notably, family disruption after age 9 seemed to be less harmful with regard to school completion than continuous single parenthood among individuals originating from primary and secondary educated families (Model 6). Among tertiary educated individuals, however, the propensity to complete secondary education was about the same (and less significant) regardless of age when the parents divorced.

A family’s socioeconomic position also affect neighbourhood of residence [36]. In the present study we found that the parental education level moderates the association with urban settlement and completion of upper secondary education. Urban settlement was associated with completion among individuals of tertiary educated families, whereas among primary educated individuals urbanity was actually associated with non-completion. The education level in rural areas is in general lower than in more urban environments [38], which may be explained partly by the social trend of selective migration among young adults [80]. School completers often move away from rural places to pursue higher education and better job opportunities. A second possible explanation for urban-rural differences in completion rates, particularly between the major cities and other municipalities, lies in the large and growing group of young people with first- or second-generation immigrant backgrounds living in urban areas. Completion rates and investment in education in these groups are weaker compared with "ethnic Norwegians" [25]. Furthermore, family SES is closely related to neighbourhood of residence and school environment [36], and families of lower SES more often settle in deprived neighbourhoods. In the countryside, however, where house prices are lower, a family with the same SES profile may settle in a neighbourhood with far better resources and facilities—which in turn has implications for whether or not young people complete secondary education. Since most individuals originating from highly educated families complete secondary education (82%), and the great majority of the Norwegian population is settled in urban areas, the high-risk group seems to be urban adolescents whose parents have low education. In light of the relative SES hypothesis [43], communities characterized with large material inequalities—which are more common in urban areas—will more severely impact those who consider themselves at a lower socioeconomic level relative to their neighbouring peers.

In summary, adverse family structural factors, which are more prevalent in families of lower education level, increase the likelihood of not completing upper secondary education. The negative effect is strongest among individuals originating from families with low educational backgrounds. Moreover, the effect of the rural-urban dimension differs among the education groups. Urban place of residence was positively associated with school completion, but only among individuals originating from tertiary educated families. These findings indicate that the key issue is likely the family´s allocation of resources. High education level is often associated with higher income, more flexible jobs, and more use of hired help, which in turn affects the children’s living environment. Furthermore, if the family is the only or the primary source of material and social resources, structure may matter more than if there are multiple sources outside the family orbit. Previous research has, for instance, demonstrated social differences in types and quality of social networks and social support [1]. Thus, two-parent and high income families not only have more time and resources to invest in their offspring, but they also tend to be embedded in social networks that facilitate the development of social and human resources in their children [19]. Families of higher SES have, in other words, more resources available at multiple levels, which dampen the impact of disadvantageous life events such as family disruption or young maternal age. Moreover, measures of additive interaction (see S4 and S5 Tables) show that the combined effect of parental unemployment and low education level on school non-completion was higher (S = 1.53 and S = 1.44) than the sum of the individual effects of the variables. Since people with low education are more likely to be unemployed, poor, and single parents, and reside in deprived neighbourhoods [1], the negative impact of low educational family origin may be quite substantial.

The connection between educational achievement and parental education level makes the individual life course seem predetermined. The interconnection and complexity of sources of inequality pose a barrier to reducing social inequalities. Norway is a relatively egalitarian country [81], but mechanisms of intergenerational transmission of social inequalities threaten this status. It is thus important to understand how social and economic resources interact and moderate these effects. The complexity of social inequalities and the generational transmissions of SES affect a range of policy arenas, including education, family (parental leave, family benefits, childcare services, etc.), employment, welfare, health, and housing.

It is reasonable to assume that the mechanisms identified here are even more powerful in countries with greater social inequalities than Norway [51]. At the same time, it has long been established that educational level is the single variable that influences people’s lives most radically towards a more prosperous life direction. Therefore public policies in Norway and elsewhere should, on basis of existing and new knowledge, focus on strengthening the investment in societies to secure a positive impact on educational inequalities across subgroups of the population. In this regard, it is important to ensure that targeted measures to improve completion rates include vulnerable groups of the population, such as families of lower education level, that often fail to take advantage of such measures. The key role of the family as a social context in the process of educational attainment should be acknowledged and emphasized to a greater extent. Future research should continue to illuminate the role of different factors in the lives of young people, which will provide the largest impact on secondary education completion rates.

### Methodological considerations

One of the major strengths of this study is the use of the large, register-based, nationally representative data set. The use of high quality, official longitudinal register-data covering almost the entire Norwegian population greatly minimizes the risk of selection bias and systematic errors. Additionally, we avoid recall bias that can affect retrospective reports of childhood conditions. All information on childhood and family characteristics come from censuses of current data, and the synthesis of this information provides a dynamic picture of childhood within the sample. Furthermore, using data from administrative registers enables us to track the same individuals over long periods of time and merge information from multiple records. In this way, we can combine information from parents, households, and subjects. Moreover, by using registry data that covers virtually the whole population, we are able to avoid problems related to non-respondents in traditional survey designs and the potential of selection bias associated with non-participation.

The study has several limitations. Perhaps the most important shortcoming is that we have no data distinguishing biological parent-child bonds from adoptive ones. Hence, this study can only view families as a social context without controlling for genetic factors. Moreover, an ideal life course study includes explanatory variables at multiple levels and allows the levels (e.g., the context) to change over time. A highly important level in the present study is the school or schools the children attended before age 16, which indeed may change during childhood. Furthermore, the child may also live in different households and neighbourhoods and adjustment for this would be strong advancement of the study. Consequently, this study should be analysed by a multiple membership cross-classified multilevel analysis that allows these levels (neighbourhoods, schools, and families) to change over time. However, our data does not include information about the school nor the particular household the child attended before age 16 (when the individual started upper secondary education) and thus prevents this analytic framework.

The methodological challenges in the analysis of neighbourhood contextual effects are discussed by others and include the identification of the appropriate boundaries [82], endogeneity, structural confounding, and multilevel regression analyses [83]. In the present study the variance at the neighbourhood level was much lower than at the family level, which is in agreement with other studies. An obvious challenge related to the investigation of neighbourhood effects in Norway is the major differences in population density between the different regions. A small proportion of the population (about 19%) lives in rural areas, which cover about 99% of Norway’s land area [66]. Since our study sample is a 30% random and stratified sample of the population, a significant proportion of the neighbourhoods include only a small number of study participants. Thus, we should be very careful drawing any causal relationship between neighbourhood and completion of secondary education. Moreover, a relevant question is also whether the enumeration districts are appropriate boundaries to embrace a relevant context that conditions individual differences in school completion.

Other confounding variables which affect both childhood conditions and completion of secondary education may be unobserved, such as factors related to personality, lifestyle, and school performance, as well as other factors related to childhood environmental characteristics not fully captured by the data. We do believe, however, that the present study, with its strengths and limitations, contributes worthy knowledge to the field of educational achievement in a life course perspective.

## Supporting information

S1 Table The effects of parental education level, family structure and neighbourhood of residence, and its interactions on the probability of completing secondary education at age 21 among individuals in family groups of more than one child (N = 16,170)–three level logistic regression models estimated by mixed effects method, STATA/MP software. (PDF)

S2 Table The effects of parental education level, family structure and neighbourhood of residence, and its interactions on the probability of completing secondary education at age 21 among individuals in family groups of more than one child (N = 16,170)–two level logistic regression models estimated by Markov chain Monte Carlo (MCMC) method, MLwiN multilevel modelling software. (PDF)

S3 Table The effects of parental education level, family structure and neighbourhood of residence, and its interactions on the probability of completing secondary education at age 21 among individuals in family groups of more than one child (N = 16,170)–two level logistic regression models estimated by mixed effects method, STATA/MP software. (PDF)

S4 Table Maternal unemployment and low parental education level as risk factors for non-completion of secondary education: Single effects of both exposures, joint effects when using one reference category, and measures of interaction on additive scale. (PDF)

S5 Table Paternal unemployment and low parental education level as risk factors for non-completion of secondary education: Single effects of both exposures, joint effects when using one reference category, and measures of interaction on additive scale. (PDF)

## References

[1] Berkman LF, Kawachi I, Glymour MM. Social Epidemiology. Second ed New York: Oxford University Press; 2014.

[2] Marmot MG, Wilkinson RG. Social Determinants of Health. Oxford: Oxford University Press; 2006.

[3] Nilsen SM, Bjorngaard JH, Ernstsen L, Krokstad S, Westin S. Education-based health inequalities in 18,000 Norwegian couples: the Nord-Trondelag Health Study (HUNT). BMC Public Health. 2012;12:998 10.1186/1471-2458-12-998 23157803 PMC3533525

[4] Marmot MG. Understanding social inequalities in health. Perspectives in biology and medicine. 2003;46(3):S9–S23. 14563071

[5] OECD. Education at a Glance 2014 OECD Indicators: OECD Publishing; 2014.

[6] Leinonen T, Martikainen P, Lahelma E. Interrelationships between education, occupational social class, and income as determinants of disability retirement. Scand J Public Health. 2012;40(2):157–66. 10.1177/1403494811435492 22312029

[7] Caspi A, Wright BRE, Moffitt TE, Silva PA. Early Failure in the Labor Market: Childhood and Adolescent Predictors of Unemployment in the Transition to Adulthood. American Sociological Review. 1998;63(3):424–51.

[8] Marmot MG, Bell R. Fair society, healthy lives. Public Health. 2012;126 Suppl 1:S4–10. 10.1016/j.puhe.2012.05.014 22784581

[9] Blane D. The life course, the social gradient and health In: Marmot MG, Wilkinson RG, editors. Social Determinants of Health. 2nd ed Oxford: Oxford University Press; 2006 p. 54–77.

[10] Case A, Fertig A, Paxson C. The lasting impact of childhood health and circumstance. J Health Econ. 2005;24(2):365–89. 10.1016/j.jhealeco.2004.09.008 15721050

[11] Björklund A, Jäntti M, Solon G. Nature and nurture in the intergenerational transmission of socioeconomic status: Evidence from Swedish children and their biological and rearing parents. The BE Journal of Economic Analysis & Policy. 2007;7(2).

[12] Leventhal T, Brooks-Gunn J. The neighborhoods they live in: The effects of neighborhood residence on child and adolescent outcomes. Psychological Bulletin. 2000;126(2):309–37. 10748645 10.1037/0033-2909.126.2.309

[13] Braveman P, Barclay C. Health disparities beginning in childhood: a life-course perspective. Pediatrics. 2009;124 Suppl 3:S163–75. 19861467 10.1542/peds.2009-1100D

[14] Wadsworth M. Health inequalities in the life course perspective. Social science & medicine. 1997;44(6):859–69. 9080567 10.1016/s0277-9536(96)00187-6

[15] Elder G, Johnson M, Crosnoe R. The emergence and development of life course theory: Springer; 2003.

[16] Elder G. The life course as developmental theory. Child development. 1998;69(1):1–12. 9499552

[17] Elder G, Rockwell R. The life-course and human development: An ecological perspective. International Journal of Behavioral Development. 1979;2(1):1–21.

[18] Elder G. Time, human agency, and social change: Perspectives on the life course. Social psychology quarterly. 1994:4–15.

[19] Uhlenberg P, Mueller M. Family Context og Individual Well-being: Petterns and Mechanisms in Life Course Perspective In: M J.T., S M.J., editors. Handbook of the Life Course (Handbooks of Sociology and Social Research). New York: Kluwer Academic/Plenum Publishers; 2003 p. 123–48.

[20] Bäckman O, Nilsson A. Pathways to Social Exclusion—A Life-Course Study. European Sociological Review. 2010.

[21] Poulton R, Caspi A, Milne BJ, Thomson WM, Taylor A, Sears MR, et al Association between children's experience of socioeconomic disadvantage and adult health: a life-course study. Lancet. 2002;360(9346):1640–5. 10.1016/S0140-6736(02)11602-3 12457787 PMC3752775

[22] Power C, Matthews S. Origins of health inequalities in a national population sample. Lancet. 1997;350(9091):1584–9. 10.1016/S0140-6736(97)07474-6 9393337

[23] Najman JM, Aird R, Bor W, O'Callaghan M, Williams GM, Shuttlewood GJ. The generational transmission of socioeconomic inequalities in child cognitive development and emotional health. Soc Sci Med. 2004;58(6):1147–58. 14723909 10.1016/s0277-9536(03)00286-7

[24] De Ridder KA, Pape K, Johnsen R, Holmen TL, Westin S, Bjorngaard JH. Adolescent Health and High School Dropout: A Prospective Cohort Study of 9000 Norwegian Adolescents (The Young-HUNT). PLoS One. 2013;8(9):e74954 10.1371/journal.pone.0074954 24086408 PMC3781164

[25] Støren LA, Helland H. Ethnicity Differences in the Completion Rates of Upper Secondary Education: How Do the Effects of Gender and Social Background Variables Interplay? European Sociological Review. 2009.

[26] Bradley RH, Corwyn RF. Socioeconomic Status and Child Development. Annual Review of Psychology. 2002;53(1):371–99. 10.1146/annurev.psych.53.100901.135233 11752490

[27] Astone NM, McLanahan SS. Family Structure, Parental Practices and High School Completion. American Sociological Review. 1991;56(3):309–20.

[28] Amato PR. Children of divorce in the 1990s: an update of the Amato and Keith (1991) meta-analysis. J Fam Psychol. 2001;15(3):355–70. 11584788 10.1037//0893-3200.15.3.355

[29] Steelman LC, Powell B, Werum R, Carter S. Reconsidering the effects of sibling configuration: Recent advances and challenges. Annual Review of Sociology. 2002:243–69.

[30] Downey DB. When Bigger Is Not Better: Family Size, Parental Resources, and Children's Educational Performance. American Sociological Review. 1995;60(5):746–61.

[31] Downey DB. Number of siblings and intellectual development: The resource dilution explanation. American Psychologist. 2001;56(6–7):497 11413873 10.1037//0003-066x.56.6-7.497

[32] Okechukwu C, Davison K, Emmons K. Changing health behaviors in a social context In: Berkman L, Kawachi I, Glymour M, editors. Social epidemiology Second ed New York, USA: Oxford University Press; 2014 p. 365–95.

[33] De Ridder KA, Pape K, Johnsen R, Westin S, Holmen TL, Bjorngaard JH. School dropout: a major public health challenge: a 10-year prospective study on medical and non-medical social insurance benefits in young adulthood, the Young-HUNT 1 Study (Norway). J Epidemiol Community Health. 2012;66(11):995–1000. 10.1136/jech-2011-200047 22315238

[34] Bruusgaard D, Smeby L, Claussen B. Education and disability pension: a stronger association than previously found. Scand J Public Health. 2010;38(7):686–90. 10.1177/1403494810378916 20709890

[35] Krokstad S, Westin S. Disability in society-medical and non-medical determinants for disability pension in a Norwegian total county population study. Soc Sci Med. 2004;58(10):1837–48. 10.1016/S0277-9536(03)00409-X 15020001

[36] Evans GW, Kantrowitz E. Socioeconomic status and health: the potential role of environmental risk exposure. Annual review of public health. 2002;23(1):303–31. 10.1146/annurev.publhealth.23.112001.112349 11910065

[37] Ainsworth JW. Why Does It Take a Village? The Mediation of Neighborhood Effects on Educational Achievement. Social Forces. 2002;81(1):117–52.

[38] Welch A, Helme S, Lamb S. Rurality and Inequality in Education International studies in educational inequality, theory and policy: Springer; 2007 p. 602–24.

[39] Bertolini P, Pisano E, Sivini S, Scaramuzzi S. Poverty and social exclusion in rural areas. POVERTY AND SOCIAL EXCLUSION IN RURAL AREAS. 2008:197.

[40] Lynch JW, Smith GD, Kaplan GA, House JS. Income inequality and mortality: importance to health of individual income, psychosocial environment, or material conditions. British Medical Journal. 2000;320(7243):1200 10784551 10.1136/bmj.320.7243.1200 PMC1127589

[41] Subramanian S, Kawachi I. Income inequality and health: what have we learned so far? Epidemiologic reviews. 2004;26(1):78–91. 15234949 10.1093/epirev/mxh003

[42] Marmot M, Wilkinson RG. Psychosocial and material pathways in the relation between income and health: a response to Lynch et al. Bmj. 2001;322(7296):1233–6. 11358781 10.1136/bmj.322.7296.1233 PMC1120336

[43] Wilkinson RG. Socioeconomic determinants of health. Health inequalities: relative or absolute material standards? BMJ: British Medical Journal. 1997;314(7080):591 9055723 10.1136/bmj.314.7080.591 PMC2126067

[44] Wilkinson RG. Ourselves and others—for better or worse: social vulnerability and inequality In: Marmot MG, Wilkinson RG, editors. Social Determinants of Health. Oxford Oxford University Press; 2006 p. 341–57.

[45] Marmot MG, Bosma H, Hemingway H, Brunner E, Stansfeld S. Contribution of job control and other risk factors to social variations in coronary heart disease incidence. The lancet. 1997;350(9073):235–9. 10.1016/s0140-6736(97)04244-x 9242799

[46] Marmot MG, Shipley MJ, Rose G. Inequalities in death—specific explanations of a general pattern? The Lancet. 1984;323(8384):1003–6. 10.1016/s0140-6736(84)92337-7 6143919

[47] Marmot MG, Stansfeld S, Patel C, North F, Head J, White I, et al Health inequalities among British civil servants: the Whitehall II study. The Lancet. 1991;337(8754):1387–93. 10.1016/0140-6736(91)93068-k 1674771

[48] van Rossum CT, Shipley MJ, van de Mheen H, Grobbee DE, Marmot MG. Employment grade differences in cause specific mortality. A 25 year follow up of civil servants from the first Whitehall study. Journal of Epidemiology and Community Health. 2000;54(3):178–84. 10.1136/jech.54.3.178 10746111 PMC1731642

[49] Winkleby M, Cubbin C, Ahn D. Effect of cross-level interaction between individual and neighborhood socioeconomic status on adult mortality rates. American journal of public health. 2006;96(12):2145–53. 10.2105/AJPH.2004.060970 17077398 PMC1698146

[50] Hales S, Howden-Chapman P, Salmond C, Woodward A, Mackenbach J. National infant mortality rates in relation to gross national product and distribution of income. The Lancet. 1999;354(9195):2047. 10.1016/S0140-6736(99)03763-0 10636372

[51] Wilkinson RG, Pickett KE. Income inequality and population health: a review and explanation of the evidence. Social science & medicine. 2006;62(7):1768–84. 16226363 10.1016/j.socscimed.2005.08.036

[52] Kawachi I, Kennedy BP, Lochner K, Prothrow-Stith D. Social capital, income inequality, and mortality. American journal of public health. 1997;87(9):1491–8. 9314802 10.2105/ajph.87.9.1491 PMC1380975

[53] Wilkinson RG. Health, hierarchy, and social anxiety. Annals of the New York Academy of Sciences. 1999;896(1):48–63. 10681887 10.1111/j.1749-6632.1999.tb08104.x

[54] Wilkinson RG. Income inequality, social cohesion, and health: clarifying the theory—a reply to Muntaner and Lynch. International Journal of health services. 1999;29(3):525–43. 10450545 10.2190/3QXP-4N6T-N0QG-ECXP

[55] Nieuwenhuis J, Hooimeijer P, Meeus W. Neighbourhood effects on educational attainment of adolescents, buffered by personality and educational commitment. Social science research. 2015;50:100–9. 10.1016/j.ssresearch.2014.11.011 25592923

[56] Krokstad S, Johnsen R, Westin S. Social determinants of disability pension: a 10-year follow-up of 62 000 people in a Norwegian county population. Int J Epidemiol. 2002;31(6):1183–91. 12540720 10.1093/ije/31.6.1183

[57] Stafford M, McCarthy M. Neighbourhoods, housing, and health In: Marmot MG, Wilkinson RG, editors. Social Determinants of Health. 2nd ed Oxford Oxford University Press; 2006 p. 297–317.

[58] Stafford M, Marmot M. Neighbourhood deprivation and health: does it affect us all equally? International Journal of Epidemiology. 2003;32(3):357–66. 12777420 10.1093/ije/dyg084

[59] Akselsen A, Lien S, Siverstøl Ø. FD-Trygd, List of variables. Oslo/Kongsvinger, Norway: Statistics Norway/Department of Social Statistics/Division for Social Welfare Statistics, 2007.

[60] SSB. Norwegian Standard Classification of Education Oslo/Kongsvinger Statistics Norway, 2001 82-537-4848-5

[61] Education—from Kindergarten to Adult Education. Norwegian Ministry of Education and Research; 2007.

[62] Chaudhary M. Sju av ti fullfører videregående opplæring. [Seven out of ten complete secondary education]. (In Norwegian). http://www.ssb.no/utdanning/artikler-og-publikasjoner/sju-av-ti-fullforer-videregaaende-opplaering: Statistic Norway, 2011 01.07.2015. Report No.

[63] Olausson PO, Haglund B, Weitoft GR, Cnattingius S. Teenage Childbearing and Long-Term Socioeconomic Consequences: A Case Study in Sweden. Family Planning Perspectives. 2001;33(2):70–4. 11330853

[64] Hauser R, Warren J, Haung M, Carter W. Social stratification across three generations In: Arrow K, Bowles S, Durlauf S, eds. Meritocracy and inequality.: Princeton, NJ: Princeton University Press; 2000 179–229 p.

[65] Mercy JA, Steelman LC. Familial influence on the intellectual attainment of children. American Sociological Review. 1982:532–42.

[66] SSB. Population and area in urban settlements 2016 [In Norwegian] [Official statistics]. http://www.ssb.no/befolkning/statistikker/beftett/aar/2016-12-06: Statistics Norway; 2016 [updated 23.01.2017]. http://www.ssb.no/befolkning/statistikker/beftett/aar/2016-12-06.

[67] Goldstein H. Multilevel Statistical Models. London: Arnold 1995.

[68] Rabe-Hesketh S. S A. Multilevel and longitudinal modeling using Stata: STATA press; 2012.

[69] Snijders T A. B., Bosker R J. Multilevel Analysis: An Introduction to Basic and Advanced Multilevel Modeling. London: Sage Publishers; 2012.

[70] Merlo J, Chaix B, Yang M, Lynch J, Råstam L. A brief conceptual tutorial of multilevel analysis in social epidemiology: linking the statistical concept of clustering to the idea of contextual phenomenon. Journal of Epidemiology and Community Health. 2005;59(6):443–9. 10.1136/jech.2004.023473 15911637 PMC1757045

[71] Knol MJ, VanderWeele TJ, Groenwold RH, Klungel OH, Rovers MM, Grobbee DE. Estimating measures of interaction on an additive scale for preventive exposures. European journal of epidemiology. 2011;26(6):433–8. 10.1007/s10654-011-9554-9 21344323 PMC3115067

[72] Rasbash J, Leckie G, Pillinger R, Jenkins J. Children's educational progress: partitioning family, school and area effects. Journal of the Royal Statistical Society: Series A (Statistics in Society). 2010;173(3):657–82.

[73] Dundas R, Leyland AH, Macintyre S. Early-life school, neighborhood, and family influences on adult health: a multilevel cross-classified analysis of the Aberdeen Children of the 1950s Study. American journal of epidemiology. 2014:kwu110. 10.1093/aje/kwu110 PMC4082339 24925065

[74] Merlo J, Viciana-Fernández FJ, Ramiro-Fariñas D, Population RGotLDotA. Bringing the individual back to small-area variation studies: a multilevel analysis of all-cause mortality in Andalusia, Spain. Social science & medicine. 2012;75(8):1477–87. 22795359 10.1016/j.socscimed.2012.06.004

[75] Browne WJ, Subramanian SV, Jones K, Goldstein H. Variance partitioning in multilevel logistic models that exhibit overdispersion. Journal of the Royal Statistical Society: Series A (Statistics in Society). 2005;168(3):599–613.

[76] Browne WJ, Rasbash J. MCMC estimation in MLwiN: Citeseer; 2009.

[77] Spiegelhalter DJ, Best NG, Carlin BP, Van Der Linde A. Bayesian measures of model complexity and fit. Journal of the Royal Statistical Society: Series B (Statistical Methodology). 2002;64(4):583–639.

[78] Lunn D, Jackson C, Best N, Thomas A, Spiegelhalter D. The BUGS book: A practical introduction to Bayesian analysis: CRC press; 2012.

[79] Helland H, Støren L. Sosial reproduksjon i yrkesfagene. Hvordan påvirker bakgrunnsfaktorer hvilken type kompetanse yrkesfagelever oppnår? [Social reproduction in vocational education programs. How does background factors affect which type of competence vocational students achieve?] (in Norwegian). Tidskrift for samfunnsforskning. 2011;52(2):151–80.

[80] Norman P, Boyle P, Rees P. Selective migration, health and deprivation: a longitudinal analysis. Social science & medicine. 2005;60(12):2755–71. 15820585 10.1016/j.socscimed.2004.11.008

[81] Mackenbach JP, Kunst AE, Cavelaars AE, Groenhof F, Geurts JJ, Health EWGoSIi. Socioeconomic inequalities in morbidity and mortality in western Europe. The lancet. 1997;349(9066):1655–9. 10.1016/s0140-6736(96)07226-1 9186383

[82] Merlo J, Ohlsson H, Lynch KF, Chaix B, Subramanian S. Individual and collective bodies: using measures of variance and association in contextual epidemiology. Journal of epidemiology and community health. 2009;63(12):1043–8. 10.1136/jech.2009.088310 19666637

[83] Oakes JM. The (mis) estimation of neighborhood effects: causal inference for a practicable social epidemiology. Social science & medicine. 2004;58(10):1929–52. 15020009 10.1016/j.socscimed.2003.08.004

