# Correction: Do family and neighbourhood matter in secondary school completion? A multilevel study of determinants and their interactions in a life-course perspective

**DOI:** 10.1371/journal.pone.0184231

**Published:** 2017-08-29

**Authors:** Arnhild Myhr, Monica Lillefjell, Geir Arild Espnes, Thomas Halvorsen

There is an error in the first sentence of the Results section in the Abstract. The correct sentence is: Completion rates were significantly higher within families with higher education level (82% in tertiary educated families vs. 67% and 56% in secondary and primary educated families respectively) and were strongly correlated within families (ICC = 39.6) and neighbourhoods (ICC = 5.7).

There are multiple errors in Table 2. For the “Neighbourhood variance (95% CI)” and “Family variance (95% CI) levels”, the proportional change in variance (PCV) and median odds ratio (MOR) values in Model 6 are incorrectly omitted. For the “Neighbourhood variance (95% CI)”, “Family variance (95% CI)”, and “Family variance >1 child*” levels, the median odds ratio (MOR) values for Models 1–6 are incorrect. Please see the corrected Table 2 here.

**Table 2:** The effects of parental education level, family structure and neighbourhood of residence, and its interactions on the probability of completing secondary education at age 21 among individuals born in the period 1983–1989.

	Model 1		Model 2		Model 3		Model 4			Model 5			Model 6		
	OR	95% CI	OR	95% CI	OR	95% CI	OR	95% CI		OR	95%CI		OR	95%CI	
**Fixed effects**															
**Individual level**															
Female	1.99	1.91–2.07	1.98	1.90–2.06	1.97	1.89–2.06	1.98	1.90–2.06		1.98	1.90–2.06		1.97	1.89–2.06	
Teenage parent	0.08	0.07–0.10	0.09	0.08–0.10	0.09	0.08–0.10	0.09	0.08–0.10		0.09	0.08–0.10		0.09	0.08–0.10	
**Family level**															
Family education level															
Primary			Ref		Ref		Ref			Ref					
Secondary	1.77	1.68–1.86	1.58	1.50–1.66	1.60	1.51–1.70	1.51	1.37–1.66		1.48	1.36–1.61		1.44	1.27–1.62	
Tertiary	4.22	3.95–4.51	3.44	3.23–3.66	3.69	3.44–3.97	3.23	2.94–3.55		3.03	2.27–3.35		2.97	2.63–3.37	
Siblings															
Only child	Ref		Ref		Ref		Ref			Ref			Ref		
2–3	1.08	1.02–1.13	1.09	1.03–1.15	1.08	1.03–1.14	1.09	1.03–1.15		1.09	1.03–1.15		1.08	1.03–1.14	
4+	0.83	0.77–0.90	1.00	0.92–1.08	0.99	0.92–1.07	1.00	0.93–1.08		1.00	0.93–1.08		1.00	0.92–1.08	
Family living situation															
Two parents at age 9 and 16	Ref		Ref		Ref		Ref			Ref			Ref		
Both parent at age 9, one at age 16	0.42	0.40–0.45	0.52	0.49–0.55	0.59	0.53–0.66	0.52	0.49–0.55		0.52	0.49–0.55		0.59	0.53–0.67	
One parent at age 9 and at age 16	0.32	0.31–0.34	0.50	0.47–0.53	0.53	0.49–0.58	0.50	0.47–0.53		0.50	0.47–0.53		0.53	0.49–0.58	
Not living with parents at age 16	0.13	0.11–0.16	0.27	0.23–0.33	0.32	0.25–0.41	0.27	0.23–0.33		0.27	0.23–0.33		0.32	0.25–0.41	
Maternal age at birth															
<20	0.42	0.39–0.47	0.53	0.48–0.58	0.53	0.48–0.58	0.61	0.52–0.72		0.53	0.48–0.58		0.60	0.51–0.71	
20–30	0.83	0.79–0.87	0.86	0.82–0.90	0.86	0.83–0.90	0.80	0.74–0.86		0.86	0.82–0.90		0.80	0.73–0.86	
30+	Ref		Ref		Ref		Ref			Ref			Ref		
Only one parent registered	0.93	0.79–1.08	0.85	0.72–0.99	0.86	0.73–1.01	0.85	0.73–1.00		0.85	0.72–0.99		0.86	0.82–0.96	
**Neighbourhood level**															
Urban settlement	0.94	0.90–0.99	0.97	0.93–1.02	0.97	0.93–1.01	0.97	0.93–1.02		0.89	0.83–0.96		0.89	0.82–0.96	
**Socioeconomic controls**															
Parental employment															
Both parents in work			Ref		Ref		Ref			Ref			Ref		
One parent in work			0.77	0.74–0.80	0.77	0.74–0.81	0.77	0.74–0.80		0.77	0.74–0.80		0.77	0.74–0.81	
None parents in work			0.65	0.60–0.70	0.65	0.60–0.70	0.65	0.60–0.70		0.65	0.60–0.70		0.65	0.60–0.70	
Poverty			0.42	0.39–0.44	0.42	0.39–0.44	0.42	0.39–0.44		0.42	0.39–0.44		0.42	0.39–0.44	
**Interactions with parental education level**															
Family education level*living situation															
2*Two parents at age 9, one at age16					0.88	0.76–1.01							0.87	0.76–1.01	
2*One parent at age 9 and at age 16					1.02	0.92–1.14							1.02	0.91–1.14	
2*Not living with parents at age 16					0.78	0.53–1.16							0.79	0.53–1.16	
3*Both parent at age 9, one at age 16					0.81	0.70–0.94							0.80	0.69–0.93	
3*One parent at age 9 and 16					0.82	0.73–0.91							0.81	0.72–0.91	
3*Not living with parents at age 16					0.66	0.42–1.06							0.67	0.42–1.07	
Family education level*maternal age															
2 *<20 years							1.09	0.98–1.21					1.09	0.97–1.21	
2 *20–30							0.83	0.68–1.03					0.83	0.67–1.03	
3 *<20 years							1.12	1.02–1.24					1.15	1.04–1.27	
3 *20–30							0.71	0.56–0.91					0.78	0.61–1.00	
Family education level*Urban															
2 *Urban										1.10	0.99–1.21		1.09	0.99–1.21	
3 *Urban										1.19	1.06–1.32		1.21	1.08–1.34	
Random effects															
**Neighbourhood variance (95% CI)**	0.13	0.11–0.16	0.11	0.09–0.13	0.11	0.08–0.13	0.11	0.08–0.13		0.11	0.08–0.13		0.10	0.08–0.13	
PCV	-60.6%		-66.7%		-66.7%		-66.7%			-66.7%			-69.7		
ICC(%)	2.7		2.23		2.23		2.23			2.21			2.21		
MOR	1.41		1.37		1.37		1.37			1.37			1.35		
**Family variance (95% CI)**	1.43	1.18–1.74	1.34	1.10–1.63	1.31	1.07–1.61	1.34	1.10–1.63		1.34	1.10–1.63		1.31	1.07–1.61	
PCV^a^	- 35.9%		- 39.9%		- 41.3%		-39.9%			- 39.9%			- 41.3%		
ICC (%)	29.5		28.3		27.9		28.3			28.3			27.8		
MOR	3.13		3.02		2.98		3.02			3.02			2.98		
Family variance >1 child*															
**Family variance (95% CI)**	1.49	1.21–1.84	1.37	1.10–1.70	1.36	1.09–1.69	1.37	1.10–1.70		1.37	1.10–1.70		1.36	1.09–1.69	
**PCV**	-34.7%		- 39.9%		- 40.4%		- 39.9%			- 39.9%			- 40.4%		
**ICC (%)**	30.6		29.0		28.8		29.0			29.0			28.8		
**MOR**	3.20		3.05		3.04		3.05			3.05			3.04		

* Family level variance from the secondary analysis containing only families with more than one child (S1 Table)

^a^The proportional change in variance expresses the change in variance at the particular level from the empty model

There is an error in S1 Table. For the “Neighbourhood variance (95% CI)” and “Family variance (95% CI)” levels, the median odds ratio (MOR) values for Models 1–6 are incorrect. Please see the corrected S1 Table here.

There is an error in S3 Table. For the “Family variance (95% CI)” level, the median odds ratio (MOR) values for Models 1–6 are incorrect. Please see the corrected S3 Table here.

## Supporting information

S1 Table The effects of parental education level, family structure and neighbourhood of residence, and its interactions on the probability of completing secondary education at age 21 among individuals in family groups of more than one child (N = 16,170)–three level logistic regression models estimated by mixed effects method, STATA/MP software. (DOCX)

S3 Table The effects of parental education level, family structure and neighbourhood of residence, and its interactions on the probability of completing secondary education at age 21 among individuals in family groups of more than one child (N = 16,170)–two level logistic regression models estimated by mixed effects method, STATA/MP software. (DOCX)
