# Supplementary material for: Do family and neighbourhood matter in secondary school completion? A multilevel study of determinants and their interactions in a life-course perspective
Source: PLoS One. 2017 Feb 21;12(2):e0172281. doi: 10.1371/journal.pone.0172281 (PMC5319759; doi:10.1371/journal.pone.0172281)
Supplement: S3 Table — (PDF) [file pone.0172281.s003.pdf]

S3 Table. The effects of parental education level, family structure and neighbourhood of residence, and its interactions on the probability of completing secondary education at age 21 among individuals in family groups of more than one child (N=16,170) – two level logistic regression models estimated by mixed effects method, STATA/MP software.

	Model 1		Model 2		Model 3		Model 4		Model 5		Model 6	
	OR	95% CI	OR	95% CI	OR	95% CI	OR	95% CI	OR	95%CI	OR	95%CI
**Fixed effects**												
**Individual level**												
Female	1.99	1.82-2.19	1.98	1.81-2.18	1.98	1.80-2.18	1.98	1.81-2.18	1.98	1.81-2.18	1.98	1.81-2.18
Teenage parent	0.09	0.07-0.13	0.10	0.07-0.13	0.10	0.07-0.13	0.10	0.07-0.13	0.10	0.07-0.13	0.10	0.07-0.13
**Family level**												
Family education level												
Primary	Ref		Ref		Ref		Ref		Ref		Ref	
Secondary	1.75	1.53-2.00	1.53	1.34-1.74	1.47	1.26-1.71	1.60	1.19-2.13	1.36	1.09-1.71	1.39	0.98-1.97
Tertiary	4.82	4.18-5.56	3.75	3.25-4.32	4.01	3.41-4.71	3.93	3.04-5.08	3.41	2.64-4.40	3.75	2.67-5.25
Siblings												
Only child	Ref		Ref		Ref		Ref		Ref		Ref	
2-3	0.91	0.55-1.51	0.87	0.52-1.44	0.85	0.51-1.40	0.88	0.53-1.46	0.87	0.52-1.45	0.86	0.52-1.42
4+	0.61	0.36-1.02	0.70	0.42-1.17	0.68	0.41-1.14	0.71	0.33-1.55	0.70	0.42-1.18	0.69	0.41-1.16
Family living situation												
Two parents at age 9 and 16	Ref		Ref		Ref		Ref		Ref		Ref	
Both parent at age 9, one at age 16	0.45	0.38-0.52	0.55	0.47-0.64	0.58	0.42-0.80	0.55	0.47-0.64	0.55	0.47-0.64	0.58	0.42-0.80
One parent at age 9 and at age 16	0.27	0.23-0.32	0.43	0.36-0.50	0.44	0.33-0.58	0.42	0.36-0.50	0.43	0.36-0.50	0.44	0.33-0.59
Not living with parents at age 16	0.17	0.09-0.31	0.33	0.18-0.59	0.40	0.19-0.87	0.33	0.18-0.59	0.33	0.18-0.59	0.41	0.19-0.87
Maternal age at birth												
<20	0.52	0.40-0.68	0.65	0.50-0.85	0.65	0.50-0.86	0.99	0.79-1.25	0.65	0.50-0.86	0.99	0.79-1.25
20-30	0.95	0.84-1.07	0.97	0.86-1.09	0.97	0.86-1.09	0.93	0.59-1.47	0.97	0.86-1.09	0.92	0.58-1.46
30+	Ref		Ref		Ref				Ref		Ref	
Only one parent registered	0.68	0.29-1.61	0.51	0.22-1.21	0.53	0.22-1.25	0.51	0.22-1.21	0.51	0.22-1.20	0.52	0.22-1.24
**Neighbourhood level**												
Urban settlement	0.92	0.82-1.03	0.95	0.85-1.06	0.95	0.85-1.06	0.95	0.85-1.06	0.85	0.68-1.06	0.85	0.68-1.05
**Socioeconomic controls**												
Parental employment												
Both parents in work			Ref		Ref		Ref		Ref		Ref	
One parent in work			0.84	0.75-0.93	0.84	0.75-0.93	0.84	0.75-0.93	0.84	0.75-0.93	0.84	0.75-0.93
None parents in work			0.69	0.55-0.85	0.69	0.56-0.85	0.69	0.56-0.85	0.69	0.56-0.85	0.69	0.56-0.86
Poverty			0.39	0.34-0.45	0.39	0.33-0.45	0.39	0.34-0.45	0.39	0.34-0.46	0.39	0.33-0.45
**Interactions with parental**												

**education level**												
Family education level*living situation												
2*Two parents at age 9, one at age16					1.15	0.77-1.71					1.16	0.78-1.74
2*One parent at age 9 and at age 16					1.17	0.82-1.67					1.16	0.82-1.66
2*Not living with parents at age 16					0.78	0.22-2.73					0.78	0.22-2.72
3*Both parent at age 9, one at age 16					0.78	0.53-1.16					0.78	0.53-1.17
3*One parent at age 9 and 16					0.77	0.53-1.11					0.76	0.53-1.10
3*Not living with parents at age 16					0.43	0.07-2.65					0.43	0.07-2.63
Family education level*maternal age												
2 *20-30							0.98	0.71-1.35			0.97	0.71-1.34
2 *30+							0.54	0.29-1.00			0.54	0.29-0.99
3 *20-30							0.96	0.72-1.28			0.97	0.73-1.30
3 *30+							0.71	0.33-1.55			0.75	0.35-1.64
Family education level*Urban												
2 *Urban									1.18	0.90-1.57	1.17	0.88-1.54
3 *Urban									1.15	0.86-1.53	1.16	0.87-1.56
Random effects												
**Family variance (95 % CI)**	1.56	1.32-1.84	1.42	1.19-1.69	1.41	1.18-1.68	1.41	1.18-1.69	1.42	1.19-1.69	1.41	1.18-1.68
**ICC (%)**	32.1		30.0		30.0		30.1		30.1		29.9	
**MOR**	3.23		3.08		3.07		3.07		3.08		3.07	
**PCV**	-37.9 %		- 43.4 %		- 43.4 %		- 43.4 %		- 43.4 %		- 43.4 %	
