# Supplementary material for: Do family and neighbourhood matter in secondary school completion? A multilevel study of determinants and their interactions in a life-course perspective
Source: PLoS One. 2017 Feb 21;12(2):e0172281. doi: 10.1371/journal.pone.0172281 (PMC5319759; doi:10.1371/journal.pone.0172281)
Supplement: S4 Table — (PDF) [file pone.0172281.s004.pdf]

S4 Table Maternal unemployment and low parental education level as risk factors for non-completion of secondary education: single effects of both exposures, joint effects when using one reference category, and measures of interaction on additive scale.

	Estimate	95 % confidence interval	
		Lower limit	Upper limit
Odds ratios representing single effects			
Maternal employment	1.0 (ref)		
Maternal unemployment	1.67	1.62	1.72
Secondary and tertiary education level (high)	1.0 (ref)		
Primary education level (low)	2.25	2.18	2.32
Odds ratios representing joint effects			
Maternal employment and high education level	1.0 (ref)		
Maternal unemployment and high education level	1.64	1.58	1.70
Maternal employment and low education level	2.20	2.12	2.28
Maternal unemployment and low education level	3.81	3.65	3.98
Measures of interaction on additive scale			
RERI	0.97		
AP	0.26		
S	1.53		
