# Supplementary material for: Do family and neighbourhood matter in secondary school completion? A multilevel study of determinants and their interactions in a life-course perspective
Source: PLoS One. 2017 Feb 21;12(2):e0172281. doi: 10.1371/journal.pone.0172281 (PMC5319759; doi:10.1371/journal.pone.0172281)
Supplement: S5 Table — (PDF) [file pone.0172281.s005.pdf]

**S5 Table** Paternal unemployment and low parental education level as risk factors for non-completion of secondary education: single effects of both exposures, joint effects when using one reference category, and measures of interaction on additive scale.

| | Estimate | 95 % confidence interval | |
|---|---|---|---|
| | | Lower limit | Upper limit |
| Odds ratios representing single effects | | | |
| Paternal employment | 1 (ref) | | |
| Paternal unemployment | 1.90 | 1.83 | 1.97 |
| High education level | 1 (ref) | | |
| Low education level | 2.26 | 2.19 | 2.33 |
| Odds ratios representing joint effects | | | |
| Paternal employment and high education level | 1 (ref) | | |
| Paternal unemployment and high education level | 1.93 | 1.84 | 2.03 |
| Paternal employment and low education level | 2.28 | 2.20 | 2.35 |
| Paternal unemployment and low education level | 4.18 | 3.94 | 4.44 |
| Measures of interaction on additive scale | | | |
| RERI | 0.97 | | |
| AP | 0.23 | | |
| S | 1.44 | | |
