# Supplementary material for: Correction: Do family and neighbourhood matter in secondary school completion? A multilevel study of determinants and their interactions in a life-course perspective
Source: PLoS One. 2017 Aug 29;12(8):e0184231. doi: 10.1371/journal.pone.0184231 (PMC5574580; doi:10.1371/journal.pone.0184231)
Supplement: S3 Table — (DOCX) [file pone.0184231.s002.docx]

**S3 Table.** The effects of parental education level, family structure and neighbourhood of residence, and its interactions on the probability of completing secondary education at age 21 among individuals in family groups of more than one child (N=16,170) – two level logistic regression models estimated by mixed effects method, STATA/MP software.

|  | Model 1 | | Model 2 | | | Model 3 | | Model 4 | | Model 5 | | Model 6 | |
| --- | --- | --- | --- | --- | --- | --- | --- | --- | --- | --- | --- | --- | --- |
|  | OR | 95% CI | OR | 95% CI | | OR | 95% CI | OR | 95% CI | OR | 95%CI | OR | 95%CI |
| **Fixed effects** |  |  |  |  | |  |  |  |  |  |  |  |  |
| **Individual level** |  |  |  |  | |  |  |  |  |  |  |  |  |
| Female | 1.99 | 1.82-2.19 | 1.98 | 1.81-2.18 | | 1.98 | 1.80-2.18 | 1.98 | 1.81-2.18 | 1.98 | 1.81-2.18 | 1.98 | 1.81-2.18 |
| Teenage parent | 0.09 | 0.07-0.13 | 0.10 | 0.07-0.13 | | 0.10 | 0.07-0.13 | 0.10 | 0.07-0.13 | 0.10 | 0.07-0.13 | 0.10 | 0.07-0.13 |
| **Family level** |  |  |  |  | |  |  |  |  |  |  |  |  |
| Family education level |  |  |  |  | |  |  |  |  |  |  |  |  |
| Primary | Ref |  | Ref |  | | Ref |  | Ref |  | Ref |  | Ref |  |
| Secondary | 1.75 | 1.53-2.00 | 1.53 | 1.34-1.74 | | 1.47 | 1.26-1.71 | 1.60 | 1.19-2.13 | 1.36 | 1.09-1.71 | 1.39 | 0.98-1.97 |
| Tertiary | 4.82 | 4.18-5.56 | 3.75 | 3.25-4.32 | | 4.01 | 3.41-4.71 | 3.93 | 3.04-5.08 | 3.41 | 2.64-4.40 | 3.75 | 2.67-5.25 |
| Siblings |  |  |  |  | |  |  |  |  |  |  |  |  |
| Only child | Ref |  | Ref |  | | Ref |  | Ref |  | Ref |  | Ref |  |
| 2-3 | 0.91 | 0.55-1.51 | 0.87 | 0.52-1.44 | | 0.85 | 0.51-1.40 | 0.88 | 0.53-1.46 | 0.87 | 0.52-1.45 | 0.86 | 0.52-1.42 |
| 4+ | 0.61 | 0.36-1.02 | 0.70 | 0.42-1.17 | | 0.68 | 0.41-1.14 | 0.71 | 0.33-1.55 | 0.70 | 0.42-1.18 | 0.69 | 0.41-1.16 |
| Family living situation |  |  |  |  | |  |  |  |  |  |  |  |  |
| Two parents at age 9 and 16 | Ref |  | Ref |  | | Ref |  | Ref |  | Ref |  | Ref |  |
| Both parent at age 9, one at age 16 | 0.45 | 0.38-0.52 | 0.55 | 0.47-0.64 | | 0.58 | 0.42-0.80 | 0.55 | 0.47-0.64 | 0.55 | 0.47-0.64 | 0.58 | 0.42-0.80 |
| One parent at age 9 and at age 16 | 0.27 | 0.23-0.32 | 0.43 | 0.36-0.50 | | 0.44 | 0.33-0.58 | 0.42 | 0.36-0.50 | 0.43 | 0.36-0.50 | 0.44 | 0.33-0.59 |
| Not living with parents at age 16 | 0.17 | 0.09-0.31 | 0.33 | 0.18-0.59 | | 0.40 | 0.19-0.87 | 0.33 | 0.18-0.59 | 0.33 | 0.18-0.59 | 0.41 | 0.19-0.87 |
| Maternal age at birth |  |  |  |  | |  |  |  |  |  |  |  |  |
| <20 | 0.52 | 0.40-0.68 | 0.65 | 0.50-0.85 | | 0.65 | 0.50-0.86 | 0.99 | 0.79-1.25 | 0.65 | 0.50-0.86 | 0.99 | 0.79-1.25 |
| 20-30 | 0.95 | 0.84-1.07 | 0.97 | 0.86-1.09 | | 0.97 | 0.86-1.09 | 0.93 | 0.59-1.47 | 0.97 | 0.86-1.09 | 0.92 | 0.58-1.46 |
| 30+ | Ref |  | Ref |  | | Ref |  |  |  | Ref |  | Ref |  |
| Only one parent registered | 0.68 | 0.29-1.61 | 0.51 | 0.22-1.21 | | 0.53 | 0.22-1.25 | 0.51 | 0.22-1.21 | 0.51 | 0.22-1.20 | 0.52 | 0.22-1.24 |
| **Neighbourhood level** |  |  |  |  | |  |  |  |  |  |  |  |  |
| Urban settlement | 0.92 | 0.82-1.03 | 0.95 | 0.85-1.06 | | 0.95 | 0.85-1.06 | 0.95 | 0.85-1.06 | 0.85 | 0.68-1.06 | 0.85 | 0.68-1.05 |
| **Socioeconomic controls** |  |  |  |  | |  |  |  |  |  |  |  |  |
| Parental employment |  |  |  |  | |  |  |  |  |  |  |  |  |
| Both parents in work |  |  | Ref |  | | Ref |  | Ref |  | Ref |  | Ref |  |
| One parent in work |  |  | 0.84 | 0.75-0.93 | | 0.84 | 0.75-0.93 | 0.84 | 0.75-0.93 | 0.84 | 0.75-0.93 | 0.84 | 0.75-0.93 |
| None parents in work |  |  | 0.69 | 0.55-0.85 | | 0.69 | 0.56-0.85 | 0.69 | 0.56-0.85 | 0.69 | 0.56-0.85 | 0.69 | 0.56-0.86 |
| Poverty |  |  | 0.39 | 0.34-0.45 | | 0.39 | 0.33-0.45 | 0.39 | 0.34-0.45 | 0.39 | 0.34-0.46 | 0.39 | 0.33-0.45 |
| **Interactions with parental**  **education level** |  |  |  |  | |  |  |  |  |  |  |  |  |
| Family education level*living situation |  |  |  |  | |  |  |  |  |  |  |  |  |
| 2*Two parents at age 9, one at age16 |  |  |  |  | | 1.15 | 0.77-1.71 |  |  |  |  | 1.16 | 0.78-1.74 |
| 2*One parent at age 9 and at age 16 |  |  |  |  | | 1.17 | 0.82-1.67 |  |  |  |  | 1.16 | 0.82-1.66 |
| 2*Not living with parents at age 16 |  |  |  |  | | 0.78 | 0.22-2.73 |  |  |  |  | 0.78 | 0.22-2.72 |
| 3*Both parent at age 9, one at age 16 |  |  |  |  | | 0.78 | 0.53-1.16 |  |  |  |  | 0.78 | 0.53-1.17 |
| 3*One parent at age 9 and 16 |  |  |  |  | | 0.77 | 0.53-1.11 |  |  |  |  | 0.76 | 0.53-1.10 |
| 3*Not living with parents at age 16 |  |  |  |  | | 0.43 | 0.07-2.65 |  |  |  |  | 0.43 | 0.07-2.63 |
| Family education level*maternal age |  |  |  |  | |  |  |  |  |  |  |  |  |
| 2 *20-30 |  |  |  |  | |  |  | 0.98 | 0.71-1.35 |  |  | 0.97 | 0.71-1.34 |
| 2 *30+ |  |  |  |  | |  |  | 0.54 | 0.29-1.00 |  |  | 0.54 | 0.29-0.99 |
| 3 *20-30 |  |  |  |  | |  |  | 0.96 | 0.72-1.28 |  |  | 0.97 | 0.73-1.30 |
| 3 *30+ |  |  |  |  | |  |  | 0.71 | 0.33-1.55 |  |  | 0.75 | 0.35-1.64 |
| Family education level*Urban |  |  |  |  | |  |  |  |  |  |  |  |  |
| 2 *Urban |  |  |  |  | |  |  |  |  | 1.18 | 0.90-1.57 | 1.17 | 0.88-1.54 |
| 3 *Urban |  |  |  |  | |  |  |  |  | 1.15 | 0.86-1.53 | 1.16 | 0.87-1.56 |
| Random effects |  |  |  |  | |  |  |  |  |  |  |  |  |
| **Family variance (95 % CI)** | 1.56 | 1.32-1.84 | 1.42 | 1.19-1.69 | | 1.41 | 1.18-1.68 | 1.41 | 1.18-1.69 | 1.42 | 1.19-1.69 | 1.41 | 1.18-1.68 |
| **ICC (%)** | 32.1 |  | 30.0 | |  | 30.0 |  | 30.1 |  | 30.1 |  | 29.9 |  |
| **MOR** | 3.29 |  | 3.12 | |  | 3.10 |  | 3.10 |  | 3.12 |  | 3.10 |  |
| **PCV** | -37.9 % | | - 43.4 % | |  | - 43.8 % | | - 43.8 % | | - 43.4 % | | - 43.8 % | |
